# Supplementary material for: Parasitic insect-derived miRNAs modulate host development
Source: Nat Commun. 2018 Jun 7;9:2205. doi: 10.1038/s41467-018-04504-1 (PMC5992160; doi:10.1038/s41467-018-04504-1)
Supplement: Supplementary file 1 — Supplementary Information [file 41467_2018_4504_MOESM1_ESM.pdf]

Parasitic insect-derived miRNAs modulate host development

Wang et al.

Supplementary Figures and Tables

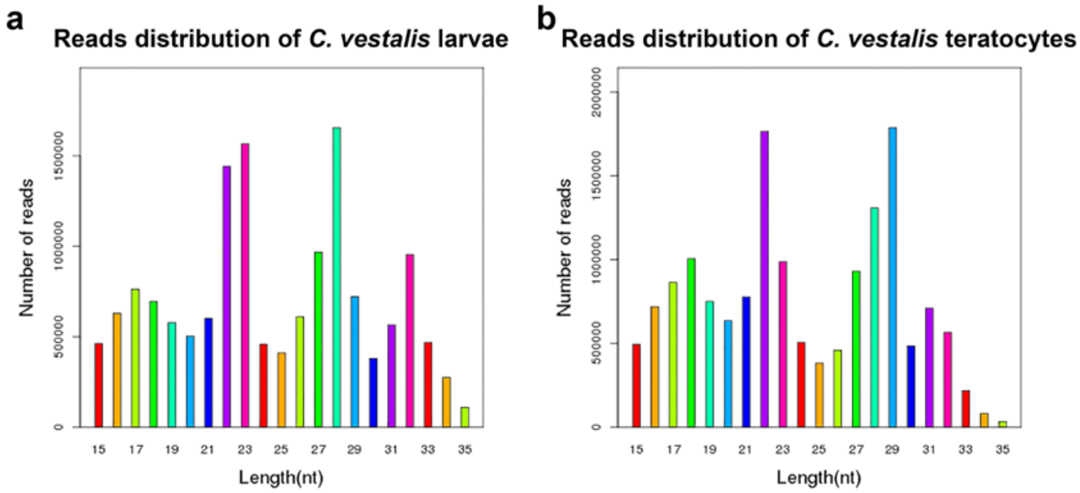

Supplementary Figure 1. Length distribution of clean reads obtained from small RNA sequencing of *C. vestalis* larvae (a) and teratocytes (b).

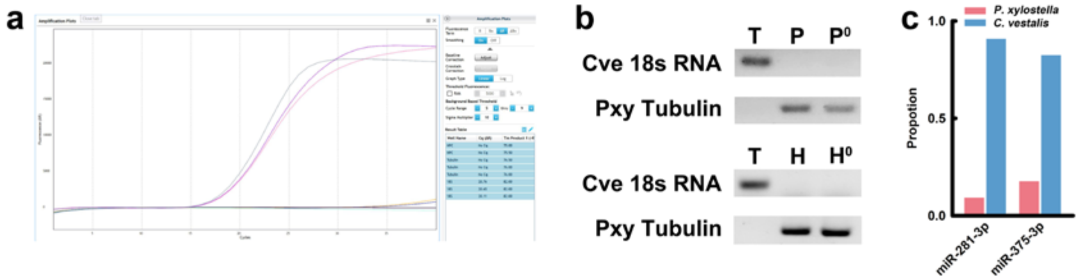

Supplementary Figure 2. Teratocytes Release miRNAs into the Host. **a** Detection of *C. vestalis 18S RNA* and *P. xylostella tubulin* by qPCR. **b** *C. vestalis 18S RNA* (Cve 18s RNA) and *P. xylostella tubulin* (Pxy Tubulin) were used as markers for assessing the purity of *P. xylostella* Pxem_ZJU cells (upper panel) or hemocytes (lower panel) used in experiments after separation from *C. vestalis* teratocytes. In the upper panel, Pxem_ZJU cells were either co-cultured with teratocytes (P) or cultured without teratocytes (P^{o}) (control). After separating Pxem_ZJU cells (P) from teratocytes (T), Cve 18S RNA was only detected in the teratocyte sample, whereas Pxy Tubulin was only detected in Pxem_ZJU cell sample. As expected, only Pxy Tubulin was detected in Pxem_ZJU cells cultured without teratocytes. In the lower panel, *P. xylostella* hemocytes were either collected from parasitized larvae (H^{o}) that also contained *C. vestalis* teratocytes (T) or non-parasitized larvae (H). After separating hemocytes from teratocytes in parasitized samples, Cve 18S RNA was only detected in the teratocyte sample while Pxy Tubulin was only detected in the hemocyte sample. As expected, only Pxy Tubulin was detected in the hemocyte sample collected from non-parasitized *P. xylostella* larvae. **c** Percentage of miR-281-3p and miR-375-3p detected in hemocytes and teratocytes after collection from parasitized larvae (*n*=100). Primers were designed based on the miR-281-3p and miR-375-3p of *C. vestalis* that did not include the last difference sequences compared with *P. xylostella*. PCR-amplified fragments were cloned and sequenced, and the difference sequence numbers were calculated.

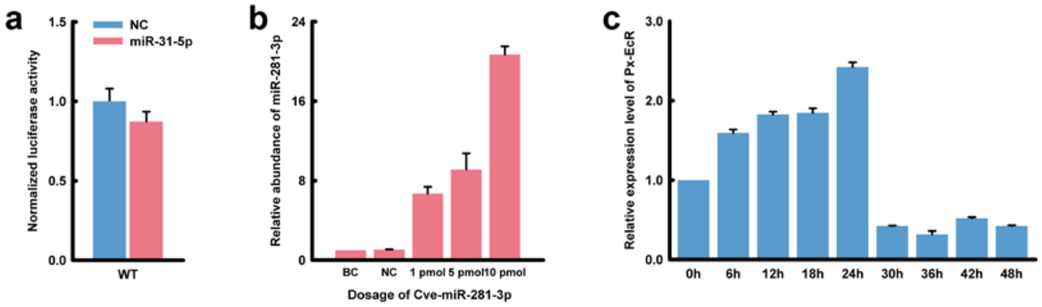

Supplementary Figure 3. miRNA function validation in vivo. **a** *In vitro* validation the target of Cve-miR-31-5p. EcR 3'-UTRs are not the direct targets of Cve-miR-31-5p. **b** miR-281-3p levels were increased by Cve-miR-281-3p mimic treatments. Different doses of Cve-miR-281-3p (1 pmol, 5 pmol and 10 pmol) were injected into the third larvae of *P. xylostella*, and the miR-281-3p levels in *P. xylostella* haemocytes were detected 12 h post injection. BC: blank control; NC: negative control mimics (5 pmol). **c** Expression level of *EcR* in third instar of *P. xylostella* larvae. Results shown are mean relative abundance ± s.e.m.

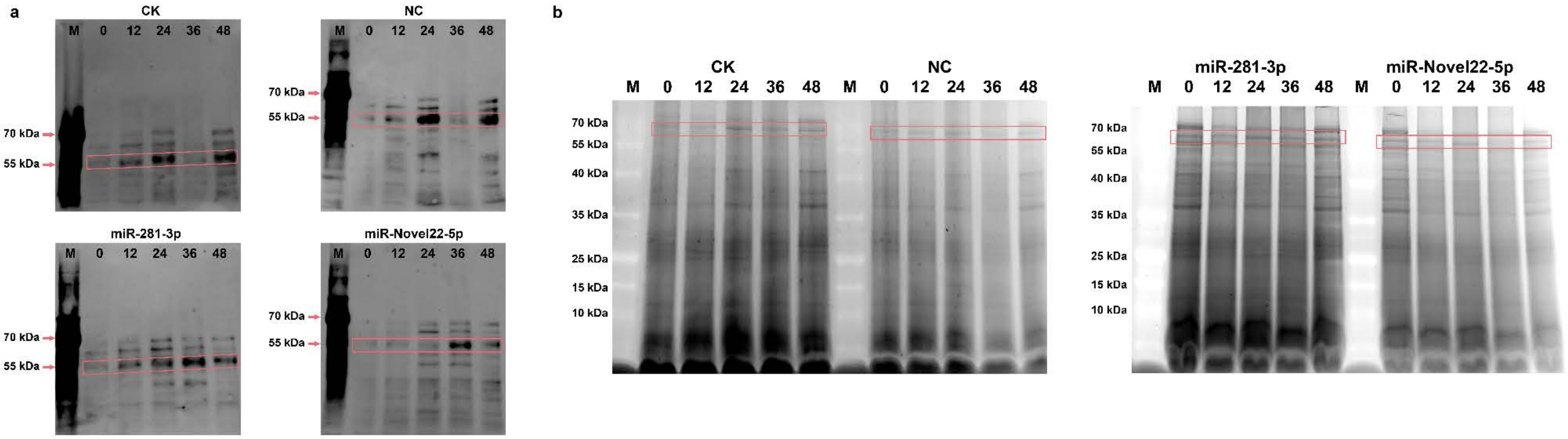

Supplementary Figure 4. The full blots or gels of Fig. 4c. a The original blots for Pxy-EcR in untreated larvae (CK), larvae injected with a control miRNA (NC), larvae injected with Cve-miR-281 or larvae injected with Cve-novel22-5p from 0 to 48 h of the third instar. western blot images were acquired using ChemiDoc-It 600 Imaging System (UVP, Upland, CA, USA). **b** The original gels of Coomassie brilliant blue G-250 (CBB) staining as a loading control of western blot. SDS-PAGE images were acquired using Densitometer GS-900 (Bio-rad, Hercules, CA, USA).

Supplementary Table 1. A summary of *C. vestalis* larvae and teratocytes small RNA data analysis

Group of reads		*C. vestalis* larvae		*C. vestalis* teratocytes	
		No. of reads	Mappable reads (%)	No. of reads	Mappable reads (%)
Mapped to *C. vestalis* genome	Mapped to miRNAs/pre-miRNAs in miRBase	4332162	37.99	1580365	10.23
	Mapped to protein coding mRNAs	289771	2.54	2778727	17.99
	Mapped to other RNAs (RFam: rRNA, tRNA, snRNA, snoRNA and others)	5359281	47.00	6649933	43.05
	Total	11403417	100.00	15447106	100.00
Unmapped to *C. vestalis* genome	Mapped to miRNAs/pre-miRNAs in miRBase	8	0.66	27737	0.71
	Mapped to tanscriptome	12	0.99	436	0.01
	Total	1217	100.00	3929007	100.00

Supplementary Table 2. Length distribution and copy number of miRNAs from *C. vestalis* larvae

Length	No. of miRNAs	%	miRNAs copy number	%
18	1	0.4854	7	0.0001
19	1	0.4854	322	0.0064
20	11	5.3398	6,272	0.1251
21	27	13.1068	1,469,878	29.3270
22	97	47.0874	2,024,640	40.3957
23	45	21.8447	1,136,889	22.6832
24	19	9.2233	299,925	5.9841
25	3	1.4563	74,065	1.4777
26	2	0.9709	25	0.0005
Total	206	100	5,012,023	100

Supplementary Table 3. Length distribution and copy number of miRNAs from *C. vestalis* teratocytes

Length	No. of miRNAs	%	miRNAs copy number	%
18	2	0.9852	1,331	0.0748
19	1	0.4926	547	0.0307
20	9	4.4335	2,797	0.1572
21	25	12.3153	642,725	36.1252
22	95	46.7980	586,805	32.9821
23	46	22.6601	520,126	29.2343
24	20	9.8522	14,018	0.7879
25	4	1.9704	10,810	0.6076
26	1	0.4926	2	0.0001
Total	203	100	1,779,161	100

Supplementary Table 4. Primers used in this paper

Primer name	Sequence (5'-3')	Usage
Pxy-EcR-F	ACACTCGTGACAACTACCGCAAG	3'UTR
Pxy-EcR-R	TGATAACACGAGGGCAAACACAAC	3'UTR
Pxy-EcR-qF	ACCTGAACACTCTCCGGGTATACAT	q-RT-PCR
Pxy-EcR-qR	AGCTTGAGTGAGATGCACATGTTG	q-RT-PCR
miR-9-SNP	TCTTTGGTCATCTAGCT	Nucleotide variation
miR-281-SNP	CTGTCATGGAATTGCTCTCTTT	Nucleotide variation
miR-375-SNP	TTTGTTCGTTCGGCTCGAG	Nucleotide variation
miR-750-SNP	AGTTGGAAGTGAGGAT	Nucleotide variation
miR-7929-SNP	TTTGTGACCGTAACAA	Nucleotide variation
Cve-miR-2552-5p	AACGACAATTTTAGGCATCTC	q-RT-PCR
Cve-miR-31-5p-1	CGTACTTGTCTTCTCTTGCGC	q-RT-PCR
Cve-miR-31-5p-2	GAGGCAAGATGTCGGCATAG	q-RT-PCR
Cve-miR-3355-3p	CAATACTATTGGGTGAAACCAA	q-RT-PCR
Cve-miR-375-3p	TTTGTTCGTTCGGCTCGAG	q-RT-PCR
Cve-miR-375-5p	CATCGATCCGGACGATCA	q-RT-PCR
Cve-miR-3811-3p	TATGTACAGTTGGACTTAGCCCT	q-RT-PCR
Cve-miR-3811-5p	TCAGCTAATTCCAGTGGACATT	q-RT-PCR
Cve-miR-750-3p	GCCAGATCTATTTCTTCCAACTC	q-RT-PCR
Cve-miR-7929-3p	GCTCACTGATACGTTCACAAGTT	q-RT-PCR
Cve-miR-7929-5p	TTTGTGACCGTAACAATGGG	q-RT-PCR
Cve-miR-9-3p-1	CGATAAAGCTAGAATACCGAAGTAA	q-RT-PCR
Cve-miR-bantam-3p-4	CGTGAGATCATTGAACAGCTAGTAT	q-RT-PCR
Cve-miR-bantam-5p-4	CGTCTAGGTGTTAAGTGATCTTATG	q-RT-PCR
Cve-miR-novel21-3p	TGAGTGAAGCAGATTCCTGATG	q-RT-PCR
Cve-miR-novel21-5p	TCAGGATCTTTGCTCTGCC	q-RT-PCR
Cve-miR-novel22-5p	TCGTATGATTACAAGGCGCT	q-RT-PCR
Cve-miR-novel23-3p	TAGCACCTTGAGATATCGAAGA	q-RT-PCR
Cve-miR-novel25-5p	TCGATTGCTTAAGAGTTTCCAT	q-RT-PCR
Cve-miR-750-3p	CCAGATCTATTTCTTCCAACTC	q-RT-PCR
Cve-miR-7929-3p	CTCACTGATACGTTCACAAGTT	q-RT-PCR
Cve-miR-309-3p	TCACTGGGAGTAATAGGTTGC	q-RT-PCR
Cve-miR-31-3p	TGGCAAGAAAAAACAATTACTG	q-RT-PCR
Cve-miR-2552-3p	TGAGGTCATAGAATTGTTGTAA	q-RT-PCR
Cve-miR-2552-5p	AACGACAATTTTAGGCATCTC	q-RT-PCR
Cve-miR-novel20-3p	ACTGTTGTTAAAGACTGCAACG	q-RT-PCR
Cve-miR-novel20-5p	TAGCTGCCTTTAACAACAAGTG	q-RT-PCR
Cve-miR-novel22-3p	TAGTACCTTCAATCATCGAAGC	q-RT-PCR
Cve-miR-novel24-3p	TAGAAACTTGGATTACTTAATA	q-RT-PCR

Continue on next page…

Primer name	Sequence (5'-3')	Usage
Cve-miR-novel24-5p	TTTAAGTACTTCGAGCTTCTTATG	q-RT-PCR
Cve-miR-375-3p	TTGTTCGTTCGGCTCGAGT	q-RT-PCR
Cve-miR-8-3p	TAATACTGTCAGGTAAAGATGTCAA	q-RT-PCR
Cve-miR-184-3p	TGGACGGAGAACTGATAAGG	q-RT-PCR
Cve-miR-275-5p-2	TCAGGTACTGAGTGACTCTGAGAA	q-RT-PCR
Cve-miR-7929-5p	TTTGTGACCGTAACAATGGG	q-RT-PCR
Cve-miR-3405-3p	TTGTGACCGTAACAATGGG	q-RT-PCR
Cve-miR-190-3p	AGATATGTTTGATATTCTTGGTAAA	q-RT-PCR
Cve-miR-bantam-3p-1	TGAGATCATTGTGAAAGCTGAT	q-RT-PCR
Cve-miR-2943-5p	TTAAGTAGTAGTGCCGTAGGTAAA	q-RT-PCR
Cve-miR-2944-3p-4	CATCACAGGCAGAGTTCTAGTTA	q-RT-PCR
Cve-miR-9-5p-1	TCTTTGGTCATCTAGCTGTATGA	q-RT-PCR
Cve-miR-14-3p	TCAGTCTTTTTCTCTCTCCTATAA	q-RT-PCR
Cve-miR-750-3p	CCAGATCTATTTCTTCCAACTCA	q-RT-PCR
Cve-miR-317-3p	TGAACACAGCTGGTGGTATCT	q-RT-PCR
Cve-miR-3791-3p	TCACCGGGTAAATGTGCTT	q-RT-PCR
Cve-miR-281-3p	TGTCATGGAATTGCTCTCTTTG	q-RT-PCR
Cve-miR-8-5p	ATCTTACCGGGCAGCATTA	q-RT-PCR
Cve-miR-309-3p	TCACTGGGAGTAATAGGTTGCA	q-RT-PCR
Cve-miR-2945-3p-1	TGACTAGAATCACACTCGTCCA	q-RT-PCR
Cve-miR-34-5p	TGGCAGTGTGGTTAGCTGGT	q-RT-PCR
Cve-miR-283-5p	AAATATCAGCTGGTAATTCTGGG	q-RT-PCR
Cve-miR-375-5p	CATCGATCCGGACGATCA	q-RT-PCR
Cve-miR-9-5p-2	CTTTGGTAATACAGCTCTATGAAA	q-RT-PCR
Cve-miR-2945-3p-2	TGACTAGATCCACACTCATTAAAA	q-RT-PCR
Cve-miR-1175-3p	GAGATTCAACTCCTCCAACTTATA	q-RT-PCR
Cve-miR-281-5p	AAGAGAGCTATCCATCGACAGT	q-RT-PCR
Cve-miR-novel23-5p	CTTGATATCTTTGAAGTGTTTGTGT	q-RT-PCR
PX-sn-U6	GGCGTGACAGGTACATATACTAA	q-RT-PCR
