# Supplementary material for: Parasitic insect-derived miRNAs modulate host development
Source: Nat Commun. 2018 Jun 7;9:2205. doi: 10.1038/s41467-018-04504-1 (PMC5992160; doi:10.1038/s41467-018-04504-1)
Supplement: Supplementary file 3 — Description of Additional Supplementary Files [file 41467_2018_4504_MOESM3_ESM.docx]

**Description of Additional Supplementary Files**

File Name: Supplementary Data 1

Description: miRNA in C. vestalis

File Name: Supplementary Data 2

Description: Target prediction of selected miRNAs
